# Emotional Word Use in Informal Carers of People Living With Dementia: Linguistic Analysis of Online Discussion Forums

**DOI:** 10.2196/32603

**Published:** 2022-06-17

**Authors:** Warren James Donnellan, Jasmine Grace Warren

**Affiliations:** 1 Department of Psychology University of Liverpool Liverpool United Kingdom

**Keywords:** dementia care, online forum, emotional language, emotional states, dementia, aging, elderly population, digital health, online health

## Abstract

**Background:**

Informal dementia care is uniquely stressful and necessitates effective methods of identifying and understanding the needs of potentially at-risk carers so that they can be supported and sustained in their roles. One such method is examining carers’ engagement in online support platforms. Research has explored emotional word use on online discussion forums as a proxy for underlying emotional functioning. We are not aware of any research that has analyzed the content of posts on discussion forums specific to carers of people living with dementia in order to examine their emotional states.

**Objective:**

We addressed the following research questions: (1) To what extent does emotional language use differ between carers of people living with dementia and noncarers? (2) To what extent does emotional language use differ between spousal and parental carers? (3) To what extent does emotional language use differ between current and former carers?

**Methods:**

We used the Linguistic Inquiry and Word Count (LIWC) program to examine emotional word use on a UK-based online forum for informal carers of people living with dementia and a discussion forum control group. Carers were separated into different subgroups for the analysis: current and former, and spousal and parental.

**Results:**

We found that carers of people living with dementia used significantly more negative, but not positive, emotion words than noncarers. Spousal carers used more emotion words overall than parental carers, specifically more negative emotion words. Former carers used more emotional words overall than current carers, specifically more positive words.

**Conclusions:**

The findings suggest that informal carers of people living with dementia may be at increased risk of negative emotional states relative to noncarers. Greater negativity in spousal carers may be explained by increased caregiver burden, whereas greater positivity in former carers may be explained by functional relief of caregiving responsibilities. The theoretical/applied relevance of these findings is discussed.

## Introduction

Informal dementia care has been described as uniquely stressful [1]. Potgieter et al [2] describe a number of characteristics that may contribute to this, including the continuous, intense, and unpredictable nature of symptoms and the extended course of dementia. Research shows that carers of people living with dementia are significantly more stressed than nondementia carers [3], and this stress is associated with poorer physical [4-6] and psychological [7,8] health. Carers are also likely to suffer declines in the availability of people to provide informal support over time [9], making them an at-risk group on physical, psychological, and social levels.

Carers are not a homogeneous group; the pattern of individual differences seen between different groups of carers is not always clear. For example, caring for an ill spouse presents different challenges than caring for an ill parent [10]. Research shows that 58% of all primary carers care for a parent, whereas 26% care for a spouse [11], and that spousal carers are more burdened by caregiving than parental carers [12]. *Dementia caregiver burden* has been associated with depression, poor physical health, and reduced quality of life [13]. Pinquart and Sorensen [12] explain that parental carers may be able to moderate care demands through alternative “distractor” roles and social activities outside the home. According to Etters et al [14], spousal carers have closer relationship ties to the care recipient, which are likely to be compromised by their growing dependency and diminished quality of communication [14-16], leading to a loss of companionship and reciprocity within the marital relationship [17]. Furthermore, spousal carers may have their own chronic health conditions [12] and may share reciprocal, fluctuating caring roles with their spouse [18], which can also lead to increased burden.

Additionally, there is a growing body of research looking at the postcaring period, most commonly former carers whose loved ones have died or been admitted to a care setting [19,20]. Longitudinal research shows that, unlike current carers, who become more depressed, former carers experience improved burden [21], quality of life, mental health, perceived health status, and social participation over time [20,22,23]. In a qualitative longitudinal study of spousal carers of people living with dementia, masked for review [24] found that most carers remain or become resilient over time, despite deteriorating health of the care recipient and care status transitions, including insitutionalization and widowhood. Bond et al [22] suggest that this could reflect the alleviation of time constraints and functional relief of caregiving responsibilities seen in former carers.

The research discussed so far suggests that carers of people living with dementia experience poorer outcomes than nondementia carers and that spousal and current carers in particular may be more at risk of negative emotional states than parental and former carers of people living with dementia. This necessitates effective methods of identifying and understanding the needs of potentially at-risk carers so that they can be supported and sustained in their roles [25]. One such method is examining carers’ engagement in online support platforms. Growing numbers of carers of people living with dementia are turning to internet-based platforms for support [26]. This may be driven in part by declines in the availability of people to provide informal support to carers over time [27]. Online platforms allow carers to access support without the need for face-to-face interaction [28], which is particularly useful for those carers who are socially isolated or physically less mobile [29]. Research shows that online support can improve the well-being of carers of people living with dementia, through reducing anxiety, depression, and increasing confidence and self-efficacy [26,30]. In a qualitative evaluation of online peer support for informal carers, masked for review [31] found that the online environment creates a unique forum within which carers exchange practical information about caring and developed friendships and a sense of belonging to their caregiving community. Online discussion forums in particular have been found to improve the quality of the relationship between the carer and the care recipient, potentially through carers learning how to better interact with the care recipient, thus reducing conflict and criticism [28].

One of the main ways in which carers interact online is through written communication. There is evidence to suggest that the content of carers’ communications may provide a window through which we can examine their underlying functioning. For example, research has shown that emotional language use is a reliable predictor of underlying social and psychological states [32]. A recent study by Vine et al [33] examined emotion vocabularies in participant-generated written speech and examined their relationships to individual differences in mood, personality, and well-being. The authors found that emotion vocabulary is associated with underlying functioning; specifically, larger negative emotion vocabulary is associated with more psychological distress and poorer physical health, while larger positive emotion vocabulary is associated with higher well-being and better physical health. Online discussion forums in particular allow us to examine emotional language use as a proxy for underlying emotional states [34,35]; for example, increased use of positive emotional words on posts may be associated with greater positivity, and increased use of negative words has been associated with greater negativity [36-38]. Online discussion forums have been used as secondary data sources in several dementia studies [26,39]. They have also been used to examine language use a proxy for mental distress [35]. However, no research has systematically analyzed the content of posts on discussion forums specific to carers of people living with dementia in order to examine their emotional states. Posts on online discussion forums are advantageous over other more traditionally used data sources as they provide a naturalistic, real-time insight into carers’ experiences that are not a potential artifact of a quantitative survey or qualitative interview schedule.

This study aims to add to the existing literature by innovatively examining emotional word use as a proxy for underlying emotional states, across spousal and parental carers of people living with dementia, current and former carers of people living with dementia, and noncarers using posts from online discussion forums using the Linguistic Inquiry and Word Count (LIWC) program [40]. Although the LIWC program has been commonly used to explore emotion analysis, to the best of our knowledge, this is the first time it has been applied to the dementia care context. By using the LIWC program to examine the proportion of emotional word use as a window onto underlying emotional states, for example, increased use of positive words indicating greater positivity [33,36-38], we may be able to facilitate better identification and understanding of the needs of potentially at-risk carers so that they can be supported and sustained in their roles [25]. We address the following research questions: (1) To what extent does emotional language use differ between carers of people living with dementia and noncarers? (2) To what extent does emotional language use differ between spousal and parental carers? (3) To what extent does emotional language use differ between current and former carers? Given that carers of people living with dementia experience poorer outcomes than nondementia carers [3-8,27] and that spousal and current carers in particular may be more at risk of negative emotional states than parental [12,14-18] and former [20-24] carers of people living with dementia, we propose the following 3 hypotheses: (1) Carers of people living with dementia will use more negative words than noncarers, (2) spousal carers will use more negative words than parental carers, and (3) former carers will use more positive words than current carers.

## Methods

### Setting

Our data were drawn from Dementia Talking Point, a UK-based online discussion forum, hosted by Alzheimer’s Society, for informal carers and people living with dementia to share experience and provide peer support [11,38]. Dementia Talking Point is well used, with 122,447 threads, 1,793,837 posts, and 73,428 users as of January 27, 2022. The forum contains a number of subforums, all of which are actively used and publicly viewable without registration.

### Procedure

We selected 3 subforums for this study on August 21, 2018: “I have a partner with dementia,” “I care for a person with dementia,” and “Younger people with dementia and their carers.” We chose these subforums as they were most relevant to the study aims and population (ie, they were most likely to contain spousal and parental, and current and former informal carers of people living with dementia). The second author manually selected the 100 most recent posts from each subforum (N=300) and collated them into a Microsoft Excel spreadsheet. In line with Lyons et al [17], a maximum of 500 words per post were read to ensure that they met the inclusion criteria. Only 18 (6%) of 300 posts were over 500 words, so they were shortened to 500 words prior to analysis.

We included original posts rather than comments on posts, and 1 post per user. For those users with repeated or multiple posts, we used their earliest entry, as subsequent posts were likely to be comments on or duplications of the first post. Posts were only included if the user disclosed that they currently or previously cared for a spouse or parent living with dementia. As this information was not readily available, we determined it from the content of the posts. For example, to determine carer type, the second author looked for references such as “my wife” and “my dad” to identify spousal and parental carers, respectively. To determine care status, the second author looked for entries such as “We’re stuck at home” (current carer) and “I miss him/her so much since they were admitted” (former carer). Both authors independently coded the data to identify carer type and care status and reached an agreement with a κ of 0.93. Where there was conflict between coding, both authors discussed cases until a consensus was reached. We originally intended to include both bereaved former carers and carers who had admitted the care recipient into a care setting, but only the latter group was present; the final sample did not include any bereaved former carers. We excluded carers of grandparents, neighbors, and friends as they were not relevant to the study aims. No further information specific to the caregiving context was available from the data set.

To identify significant effects of group, carer type, and care status on emotional word use (*η*_p_^2^=0.5), a power calculation using G*Power [41] indicated that the required sample size for 95% power with *α*=.05 was N=105 per condition. Using this method, we identified 270 carers. Finally, 100 control group posts were selected from an online personal finance discussion forum [35]. The finance discussion forum we used had been used as a control group in a previous linguistic analysis [35] and was appropriate here due to the relatively low likelihood of containing carers or extremes of emotion. To improve the robustness of the control group further, control entries were excluded if the user disclosed that they currently or previously cared for a person living with dementia (Table 1).

**Table 1:** Frequencies of participant demographic characteristics.

Participants		Frequency, n (%)
Noncarers (control)		100 (27)
Carers		270 (73)
**Spousal**		119 (43)
	Current	84 (71)
	Former	31 (26)
	Missing	4 (3)
**Parental**		151 (55)
	Current	59 (39)
	Former	84 (56)
	Missing	8 (5)
**Total**		
	Current	143 (55)
	Former	115 (45)

### Data Analysis

We used the LIWC program [40] to analyze the discussion forum posts. The LIWC program is designed to capture people’s underlying social and psychological states by assessing the emotional, cognitive, and structural components of text based on a psychometrically validated dictionary of over 6400 words [42].

We examined percentage emotional word use (dependent variable; affective processes, positive, negative, anxiety, anger, sadness) across group (independent variable 1; carers and noncarers), carer type (independent variable 2; spousal and parental), and care status (independent variable 3; current and former). Affective process words encapsulated all emotion words of different valences (eg, happy, ugly, bitter). These were included as an overarching measure of linguistic emotionality. Positive words included words such as happy, pretty, and good. Negative words included words such as hate, worthless, and enemy. Anxiety words included words such as nervous, afraid, and tense. Anger words included words such as hate, kill, and annoyed. Finally, sadness words included words such as grief, cry, and sad [40]. We used negative, but not positive, emotion subdimensions (ie, anxiety, anger, sadness) for 2 reasons: First, positive emotion subdimensions were not available on the LIWC database, and second, to reflect the fact that dementia care has been shown to be uniquely stressful [1], including a wide variety of negative emotion subdimensions allowed us to capture the negative impact of dementia care more comprehensively. Although it is apparent from Table 1 that the distribution of current and former carers is different in spousal and parental carers, which may warrant further investigation, we did not conduct additional subgroup analyses as the subgroup sizes were small and uneven (eg, n=84, 71%, spousal carers were currently providing care as opposed to only n=31, 26%, formerly providing care), compromising statistical power. Furthermore, we did not control for the influence of carers’ individual characteristics on emotional word use as relevant carer demographic information was not available from the naturalistic data set.

In Table 2, we include some adapted example quotes to illustrate how emotion words typically appeared in selected carers’ posts. In line with our ethics approach (see later), these quotes have been adapted from the original posts to maintain the anonymity and confidentiality of the users. All statistical analyses were conducted using SPSS Statistics V25 (IBM Corporation) using multivariate analyses of variance (MANOVA) and Welch *F* tests.

**Table 2:** Example adapted quotes to illustrate how emotion words appeared in carers’ posts.

Emotion words	Adapted example quotes from carer posts
Affective processes^a^	“My Dad was the *kindest* man I have ever met. Now he can be *nasty* and sometimes I get *frightened*.”
Positive	“…we’ve been the *lucky* ones really.”
Negative	“My husband has vascular dementia. The past year has been *hellish*.”
Anxiety	“Lately I’m feeling *scared* and bewildered…”
Anger	“Now he is verbally *abusive*…”
Sadness	“…I get *teary* when I’m on my own.”

^a^“Affective processes” is an overarching category including positive, negative, anxiety, anger, and sadness words.

### Ethical Considerations and Governance

This study was approved for partnership within the Alzheimer’s Society Research Partnerships program. Ethical approval was not sought for the following reasons: Posts on Dementia Talking Point are publicly viewable without registration; under clause 5.4 of the “Terms and Conditions of Use” of Dementia Talking Point, users of Dementia Talking Point consent to their posts being accessed by researchers; under clause 5.3, users have the opportunity for their posts not be included in research; we do not present direct quotations from users; we only include the percentage emotional word use, and no identifying information can be ascertained from these percentages, so the data remain fully anonymous and confidential. As this is a secondary data study, we will not be interacting with the forum users in any way. Finally, according to the Economic and Social Research Council (ESRC) Framework for Research Ethics, online forums “that are intentionally” public may be considered “in the public domain” [38].

## Results

### Descriptive Statistics

This study aims to analyze emotional word use of spousal and parental, and current and former carers of people living with dementia and noncarers using posts from an online discussion forum.

Data were analyzed using 2 MANOVA: 1 for the effect of group (carer and noncarer) on emotional word use and 1 for the effect of carer type (spousal and parental) and care status (current and former) on emotional word use. Levene and Box tests indicated that the assumption of homogeneity of variance and equality of covariance (*P*<.001) had been violated. Therefore, *F* values were calculated using Welch *F* tests. As data were skewed, a log transformation was conducted on all variables following an analysis of descriptive statistics (see Table 3).

**Table 3:** Descriptive statistics^a^ for effect of group, carer type, and care status on emotional word use (values are mean and SD).

Participants			Emotional word types, mean (SD)					
		Affective processes		Positive	Negative	Anxiety	Anger	Sadness
**Group**								
	Carers	5.41 (2.63)		2.94 (2.17)	2.39 (1.76)	0.56 (0.97)	0.31 (0.59)	0.74 (1.04)
	Noncarers	3.52 (2.09)		2.55 (1.81)	0.91 (0.89)	0.17 (0.31)	0.13 (0.29)	0.26 (0.46)
**Carer type**								
	Spousal	5.56 (2.94)		2.69 (2.17)	2.82 (2.00)	0.69 (1.27)	0.39 (0.73)	0.77 (1.12)
	Parental	5.20 (2.32)		3.07 (2.10)	2.04 (1.46)	0.43 (0.63)	0.24 (0.44)	0.73 (.98)
**Care status**								
	Current	4.72 (2.11)		2.28 (1.65)	2.37 (1.56)	0.58 (0.76)	0.38 (0.70)	0.48 (.82)
	Former	6.10 (2.49)		3.66 (2.25)	2.38 (1.81)	0.43 (0.59)	0.23 (0.42)	1.10 (1.20)

^a^Non-log-transformed descriptive statistics are presented for illustrative purposes.

### Effect of Group on Emotional Word Use

**Figure 1:**
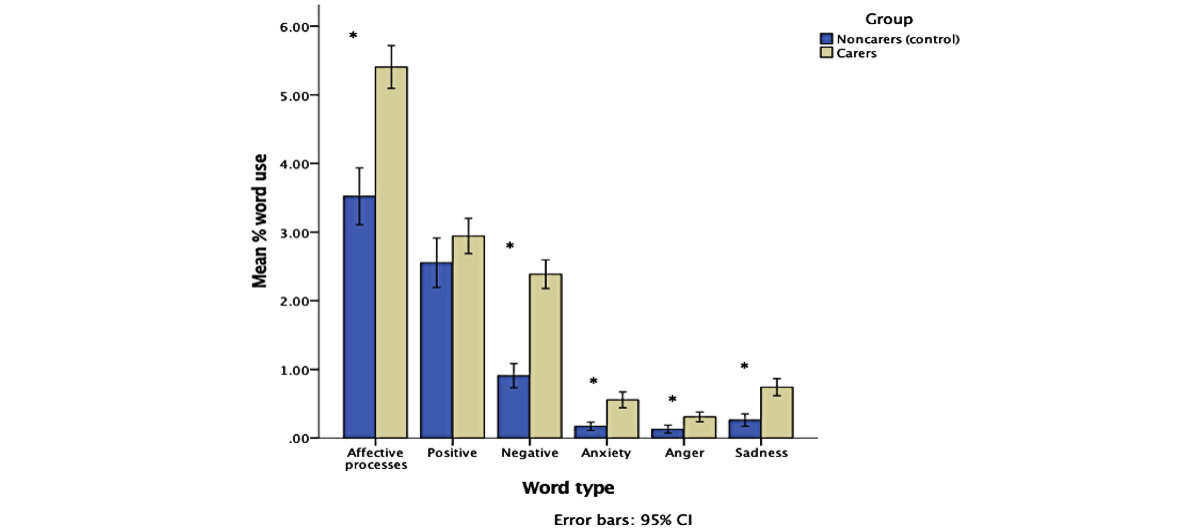
Clustered bar chart showing the effect of group on emotional word use (*P<.05). Error bars represent 95% CIs. Non-log-transformed data used for illustrative purposes.

There was a significant large effect of group on emotional word use: Pillai trace=0.22, *F*_(7, 366)_=14.49, *P*<.001, *η*_p_^2^=0.22. Welch *F* tests revealed that carers used significantly more affective process (*F*_(1,153.46)_=48.16, *P*<.001), negative (*F*_(1, 205.97)_=101.32, *P*<.001), anxiety (*F*_(1, 316.58)_=40.97, *P*<.001), anger (*F*_(1, 277.69)_=14.23, *P*<.001), and sadness (*F*_(1, 287.35)_= 34.96, *P*<.001) emotion words than noncarers. There was no difference between carers and noncarers in the use of positive emotion words (*F*_(1, 180.13)_=2.24, *P*=.14). See Figure 1.

### Effect of Carer Type and Care Status on Emotional Word Use

**Figure 2:**
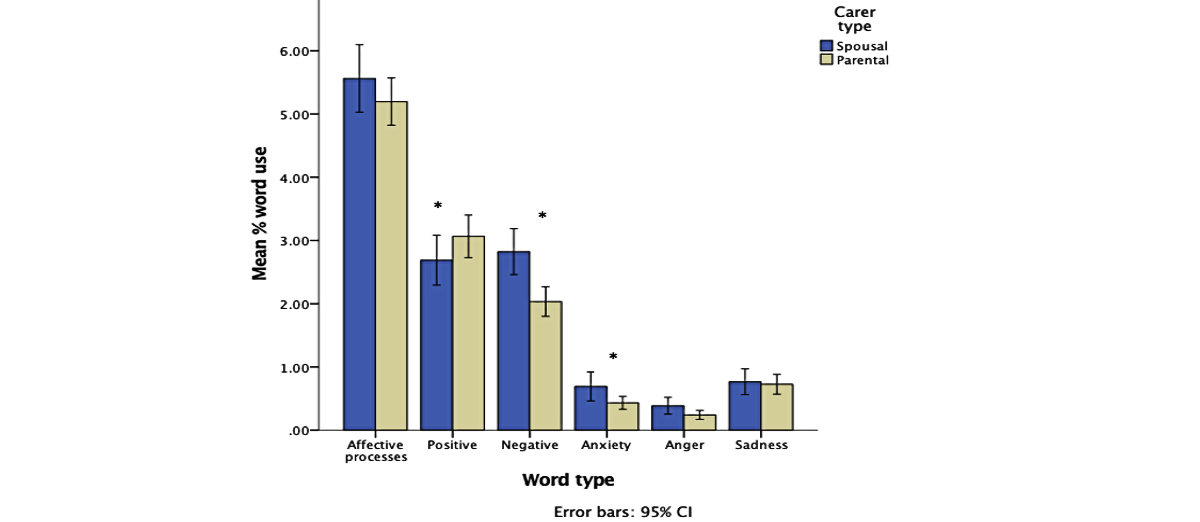
Clustered bar chart showing the effect of carer type on emotional word use (*P<.05). Error bars represent 95% CIs. Non-log-transformed data used for illustrative purposes.

There was a significant medium effect of carer type on emotional word use: Pillai trace=0.07, *F*_(7, 248)_=2.75, *P*=.01, *η*_p_^2^=0.07. Welch *F* tests revealed that spousal carers used significantly more negative (*F*_(1, 242.53)_=11.23, *P*=.001) and anxiety (*F*_(1, 215.88)_=4.33, *P*=.04) emotion words than parental carers. Parental carers used significantly more positive emotion words than spousal carers (*F*_(1, 221.67)_=5.12, *P*=.03). There was no difference between spousal and parental carers in the use of affective process (*F*_(1, 232.60)_=.51, *P*=.48), anger (*F*_(1, 207.35)_=2.56, *P*=.11), or sadness (*F*_(1, 240.15)_=.01, *P*=.92) emotion words. See Figure 2.

**Figure 3:**
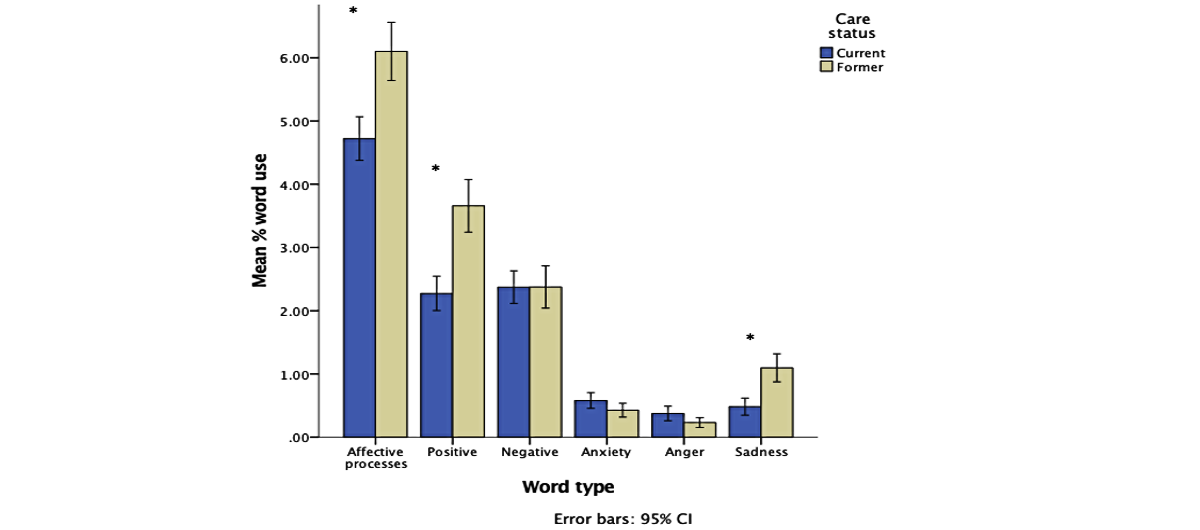
Clustered bar chart showing the effect of care status on emotional word use (*P<.05). Error bars represent 95% CIs. Non-log-transformed data used for illustrative purposes.

There was a significant large effect of care status on emotional word use: Pillai trace=0.25, *F*_(7, 248)_=11.72, *P*<.001, *η*_p_^2^=0.25. Welch *F* tests revealed that former carers used significantly more affective process (*F*_(1, 246.79)_=20.70, *P*<.001), positive (*F*_(1, 256.72)_=39.52, *P*<.001), and sadness (*F*_(1, 204.09)_=25.54, *P*<.001) emotion words than current carers. There was no difference between current and former carers in the use of negative (*F*_(1, 212.56)_=0.61, *P*=.44), anxiety (*F*_(1, 256.81)_=2.73, *P*=.10), or anger (*F*_(1, 255.59)_=2.98, *P*=.09) emotion words (Figure 3). Finally, there was no significant interaction between carer type and care status on emotional word use: Pillai trace=0.04, *F*_(7, 248)_=1.39, *P*=.21, *η*_p_^2^=0.04.

## Discussion

### Principal Findings

This study is the first of its kind to innovatively identify the extent to which online emotional language use differs between different groups of carers of people living with dementia, including noncarers. By examining posts on Dementia Talking Point, we were able to access naturalistic, carer-initiated, and real-time data from potentially unrepresented carers who may not take part in traditional research [43]. This provides an unbiased insight into the carers’ emotional states, which may enable better identification and understanding of the needs of potentially at-risk carers so that they can be better supported in their roles [25].

In line with the first hypothesis, we found that carers of people living with dementia used more affective process, negative, anxiety, anger, and sadness words, but not more positive words, than noncarers. Given that increased use of negative words indicates greater negativity [36-38], our findings suggest that informal carers of people living with dementia may be at increased risk of negative emotional states relative to noncarers. This is perhaps unsurprising and may reflect the fact that dementia carers experience unique stressors [1,2], which may negatively impinge on their psychological health [3,7,8].

We know that carers are not a homogeneous group and that there are individual differences in response to caregiving stressors. However, the direction of these individual differences has not always been clear in the literature. We demonstrate clear differences in emotional word use across both carer type and carer status, suggesting that some groups of carers are more at risk of negative emotional states than others. For example, we found that spousal carers of people living with dementia used more negative and anxiety words than parental carers, whereas parental carers used more positive words. The carers did not differ in their use of affective process, anger, or sadness words. This suggests that spousal carers are, at least in part, more negative than parental carers, which offers partial support to the second hypothesis. Our spousal carers may be relatively more negative and anxious due to the increased burden experienced by this group compared to parental carers [12,13]. This may reflect a loss of companionship and reciprocity within the marital relationship [17], which is compounded by the fact that spousal carers typically have closer relationship ties to the care recipient than parental carers [14]. Conversely, parental carers may be more positive because they are more likely than spousal carers to have alternative roles and social activities to moderate the impact of caregiving stress on their emotional states [12]. These findings have novel implications for supportive services for spousal carers of people living with dementia. The findings suggest that spousal carers are potentially more at risk of negative emotional states than parental carers and therefore may need to be prioritized, identified, and supported to sustain them in their role [25]. Specifically, spousal carers’ increased use of negative emotional language could be used by online discussion forums as a form of risk filter to provide targeted, tailored, and timely support to carers who otherwise may not have presented for support themselves [30]. Indeed, online peer support settings may be a suitable forum within which these at-risk spousal carers can be supported, not just by the service itself, but also through conversations with fellow spousal carers who may be best placed to share lived experience, advice, and guidance [31]. Research shows that this online support could potentially improve the well-being of these spousal carers by reducing anxiety and depression, increasing confidence and self-efficacy [26], and enhancing the quality of the relationship between carer and care recipient [28].

Finally, we found that former carers of people living with dementia used more affective process, positive, and sadness words than current carers. Carers did not differ in their use of negative, anxiety, or anger words. This suggests that former carers are more positive than current carers, which supports the third hypothesis. Former carers may be more positive than current carers because the people living with dementia have been admitted into a care setting, resulting in functional relief of caregiving responsibilities [22]. This may enable the carer to pursue things that were previously difficult, such as hobbies, interests, and social activities [24]. We do not suggest that former carers are entirely positive; indeed, our findings show that former carers were more emotional overall, particularly sadder, than current carers. This may reflect the sense of loss or “void” left by the care recipient after they have moved into a care setting [20]. Again, these findings are of importance to supportive service providers of current and former carers of people living with dementia. If we assume that increased positive language use in former carers indicates increased positivity, then our findings suggest that the postcaring period is not exclusively a time of negativity. Carer services, which are typically problem focused [44], should move away from a deficit approach and instead aim to routinely assess and promote the more rewarding aspects of caregiving. For example, if humor and peer support are shown to facilitate positivity in former carers, then support providers may wish to adopt an assets-based approach that celebrates the resources that carers possess rather than risk factors, challenges, and barriers alone. Indeed, recent research has shown that promoting humor and uplifts amongst an online caregiving community promotes a sense of hope and optimism [45]. Taken together, the differential patterns of emotional language and emotional states found between spousal, parental, current, and former carers reinforce the diverse heterogeneity of the caregiving population and the fact that there is no one-size-fits-all solution for support services [45]. Instead, services need to be tailored to the specific needs and circumstances of different carer types and care statuses.

### Limitations

Although this study is strengthened by its innovative methodological approach, there are a number of limitations to using such methods that need to be addressed. First, by using just 1 online discussion forum, our sample comprised only carers with internet access and those who were aware of the Dementia Talking Point website [46]. Furthermore, we could only ascertain limited care demographic data; data such as care duration, gender, and time since the care recipient was admitted into a care setting would have allowed us to better understand our findings. It was apparent that the distribution of current and former carers was different in spousal and parental carers, which may have warranted further investigation. However, the relatively small and uneven subgroup sizes precluded us from conducting additional subgroup analyses and may actually reflect real-world demographic characteristics [11]. Finally, due to ethical reasons, we had limited sociodemographic information available for users of the online personal finance discussion forum we used as our control group. Variation in their sociodemographic characteristics may have unknowingly influenced our findings relating to the first hypothesis. Furthermore, if the finance forum was being used to discuss financial systems, there may have been limited opportunities for emotional word use. If being used to seek support for financial difficulties, the use of negative emotion words may have been inflated relative to a general population. Although secondary data are useful, they pose methodological constraints [47], and this somewhat limits the generalizability of our findings to the carer population.

Although the LIWC program is useful in that it allows us to analyze percentage word use, it does not consider applied meaning or contextual information. It may also make errors in identifying and counting individual words [42]. This may have resulted in the emotional valence of some words being misinterpreted, which could have affected our findings. Fortunately, this is not likely, as the LIWC program uses probabilistic models of language use [42], but future work could adopt qualitative analysis to complement the quantitative LIWC analysis; this mixed method approach would allow more in-depth analysis, which could be triangulated to ensure the rigor of the findings.

Second, we originally intended to include a variety of former carers in our sample, including bereaved former carers. However, the 100 most recent posts that we selected did not include bereaved former carers. The emotional state of bereaved former carers is likely to be different from those who have admitted the care recipient into a care setting [24], so our findings cannot be generalized to all former carers.

Finally, this was a cross-sectional study based on discussion board posts at a given point in time. We were therefore unable to capture any changes in carers’ emotional states over time. This is problematic, given that carers of people living with dementia experience changes in well-being over time [20-24]. The cross-sectional design also precluded us from examining whether the users’ posts were met with support from other users. Future research should adopt longitudinal methods to examine changes in language use over time, with specific reference to how language use elicits certain patterns of online support.

### Conclusion

An analysis of emotional language use on online discussion forums indicates that carers of people living with dementia may be at increased risk of negative emotional states relative to noncarers. Spousal carers may be more negative than parental carers, and former carers may be more positive than current carers. Although further mixed method research using more representative samples of carers with more sociodemographic information is required, our findings are novel and have important implications that could be of interest to supportive services in general and internet-based support platforms in particular.

## Abbreviations

LIWC: Linguistic Inquiry and Word Count
